# Physicochemical Features and Peculiarities of Interaction of AMP with the Membrane

**DOI:** 10.3390/ph14050471

**Published:** 2021-05-17

**Authors:** Malak Pirtskhalava, Boris Vishnepolsky, Maya Grigolava, Grigol Managadze

**Affiliations:** Ivane Beritashvili Center of Experimental Biomedicine, Tbilisi 0160, Georgia; b.vishnepolsky@lifescience.org.ge (B.V.); maia.grigolava@science.org.ge (M.G.); gmngdz@gmail.com (G.M.)

**Keywords:** antimicrobial peptides, database, antimicrobial activity, synergism, physicochemical features

## Abstract

Antimicrobial peptides (AMPs) are anti-infectives that have the potential to be used as a novel and untapped class of biotherapeutics. Modes of action of antimicrobial peptides include interaction with the cell envelope (cell wall, outer- and inner-membrane). A comprehensive understanding of the peculiarities of interaction of antimicrobial peptides with the cell envelope is necessary to perform a rational design of new biotherapeutics, against which working out resistance is hard for microbes. In order to enable de novo design with low cost and high throughput, in silico predictive models have to be invoked. To develop an efficient predictive model, a comprehensive understanding of the sequence-to-function relationship is required. This knowledge will allow us to encode amino acid sequences expressively and to adequately choose the accurate AMP classifier. A shared protective layer of microbial cells is the inner, plasmatic membrane. The interaction of AMP with a biological membrane (native and/or artificial) has been comprehensively studied. We provide a review of mechanisms and results of interactions of AMP with the cell membrane, relying on the survey of physicochemical, aggregative, and structural features of AMPs. The potency and mechanism of AMP action are presented in terms of amino acid compositions and distributions of the polar and apolar residues along the chain, that is, in terms of the physicochemical features of peptides such as hydrophobicity, hydrophilicity, and amphiphilicity. The survey of current data highlights topics that should be taken into account to come up with a comprehensive explanation of the mechanisms of action of AMP and to uncover the physicochemical faces of peptides, essential to perform their function. Many different approaches have been used to classify AMPs, including machine learning. The survey of knowledge on sequences, structures, and modes of actions of AMP allows concluding that only possessing comprehensive information on physicochemical features of AMPs enables us to develop accurate classifiers and create effective methods of prediction. Consequently, this knowledge is necessary for the development of design tools for peptide-based antibiotics.

## 1. Introduction

Antimicrobial peptides (AMPs) can play an important role in a novel class of biotherapeutics. A common feature of AMP is the capability to interact selectively with the microbial envelope; the composition and morphology of cell envelopes vary from microbe to microbe. AMP targets for interaction can include many different molecules or molecular complexes [1,2]; some AMPs even have several targets. Moreover, the type of target changes depending on pH, salt concentration, and other environmental factors [2]. Consequently, although AMPs, as membrane-active peptides, possess properties such as amphipathicity and positive charge, the existence of different targets at the plasma membrane and cell wall explains the wide spectrum of physicochemical features expressed by AMP amino acid sequences. Indeed, amino acid sequences and 3D structures of AMPs are highly variable [2,3]. AMPs are not designed by nature to interact with a specific target, and as a consequence, there is no single mechanism of action. The variety of AMPs and their capability to fulfill their function through many different modes of action provides clues to solving easy ways to develop resistance by microbes [4]. Consequently, AMPs have become attractive to combat multidrug resistance threats [1,2]. The variety of physicochemical properties of AMP and their modes of action is profitable to deal with the problem of resistance. However, at the same time, it makes it difficult to understand the modes of action that would allow the creation of accurate tools for classification (including machine learning) and to perform de novo design of antimicrobials with required properties [3]. In order to allow for a de novo design with low cost and high efficiency, in silico predictive models have to be invoked [5]. To develop an efficient predictive model, comprehensive knowledge of the mechanisms of action of AMPs is required. This knowledge will allow us to encode amino acid sequences expressively and will aid in constructing an accurate classifier. Below, we overview the current knowledge on amino acid sequences, secondary structures, physicochemical properties, and modes of action of AMPs in order to assess the relevance of presently used classifications.

## 2. Interaction with Envelope

Interaction with an envelope, as a rule, begins by binding to the outer layer of the envelope, which can be followed by either inhibition of the vital pathways in the outer layer of the envelope or by passing through it and reaching the plasma membrane. Taking into consideration the morphology of cell walls of Gram-positive bacteria and fungi, it is supposed that the majority of AMPs can overcome the outer layer barrier of these organisms and the plasma membrane is the main target for peptides [6]. In the case of Gram-negative bacteria, the outer barrier is also a lipid bilayer. Therefore, it is not surprising that studies of the mechanisms of action of AMPs have mainly concentrated on the exploration of the modes of interactions of AMPs with membranes (artificial or natural) [7]. Conventionally, when discussing modes of action, one really implies the results of interaction of AMPs with the membrane, which is a key factor in the action; knowledge on AMP- membrane interaction is currently the most comprehensive.

The capability of the peptide to associate to a particular region or to pass through a membrane is determined by the peptide’s physicochemical features (expressed by its amino acid sequence) and by the composition of the lipid bilayer. Peptides’ concentration is also an important factor that can determine the results of AMP–membrane interaction [8]. The particular peptide may change its behavior depending on its concentration or the bilayer’s composition. For instance, cell-penetrating peptides at a certain concentration acquire antimicrobial potency [9]; therefore, effective concentration on the membrane surface determines AMP’s ability to cause perturbation in the three-dimensional structure of lipid bilayer. As a rule, the composition of phospholipids determines the type of phases and three-dimensionally ordered structures of a bilayer [10], and so it influences the behavior of AMP. The composition and structure of the cell wall can be a determinant of AMP’s concentration on the lipid bilayer surface as well, and, consequently, of the mode of interaction with the plasma membrane [7].

Different steps of the interaction with the envelope can be distinguished. In the case of cationic AMPs, these steps are: attachment to the cell wall (Gram-negative bacteria, fungi) or outer membrane (Gram-negative bacteria) surface by electrostatic interactions; reaching the plasmatic membrane and insertion into the lipid bilayer because of hydrophobicity [6]; self-aggregation or forming of the aggregates with lipids in the case of peptides with the propensity to aggregation in the lipid environment [11,12,13]; and finally, appearance of defects (permanent or transient) in the morphology of membrane with accompanying leakage, or reversible (weak) changes and passing through the lipid bilayer without leakage [14,15].

AMP’s physicochemical features and the compositions of the cell wall or outer membrane determine AMP’s concentration on the plasma membrane surface and mode of interaction with the membrane [7]. Therefore, the subtle balance between physicochemical properties of peptides and compositions of the cell wall and lipid bilayer (outer and/or inner membrane) determines the mode of action of AMP. Studies of the relationships between AMP’s physicochemical properties (PCP), compositions of the cell wall and/or lipid bilayer, and results of interaction with the envelope have uncovered many aspects of the mechanisms of action. In this review, we survey the achievements in understanding the peculiarities of interactions of antimicrobial peptides with the lipid bilayers and reveal the tasks that should be solved to get a comprehensive explanation of the mechanisms of action that could be applied in the designing of AMPs.

## 3. Physicochemical Properties

Plasmatic membranes can be considered as the main target of AMP at the cell envelope. Generally, the major factors that determine the mode of AMP interaction with membrane are physicochemical properties (PCP) of peptides, which reflect peculiarities of the amino acid composition, distribution of hydrophilic and hydrophobic residues along the chain, and 3D structure. The peculiarities of amino acid sequences provide flexibility and structural adaptability of AMPs, which are responsible for different modes of action on the membrane bilayers. Nevertheless, other determinants such as the peptide concentration and the physicochemical properties of the membrane [16] should also be considered. Consequently, a delicate balance of physical interactions with the membrane is responsible for the mode of action of the peptide. In any case, knowledge of the physicochemical properties of a peptide is essential to understand modes of interaction with membrane and to predict the results of these interactions.

### 3.1. Amino Acids Composition and Distribution

Amino acid composition can give valuable information on physicochemical features, such as the hydrophobicity and charge of peptides. Another valuable feature of AMP is amphipathicity, which requires knowledge of sequence or even structure. It is important to know the peculiarities of amino acid composition and residue distributions in the amino acid sequences of AMPs. To assess the composition and distribution of amino acids in different sets of AMPs, data from the DBAASP database [17] was explored. When looking for the peculiarities of AMP sequences, it is reasonable to use the data on peptides that are under evolutionary pressure, meaning that only ribosomal peptides should be used.

#### 3.1.1. Composition

To reveal the functionally valuable peculiarities of AMPs’ amino acid composition, the assessments have to be compared with the assessments of an “average” protein, which is a protein with an indefinite function. For the composition of an average protein, the amino acid composition from the UniProt database [18] was used. The latter database is a repository of proteins of many different functions, and therefore it can be supposed that the amino acid composition corresponds to an “average” protein where an impact of evolutionary pressure has been smoothed.

**Figure 1:**
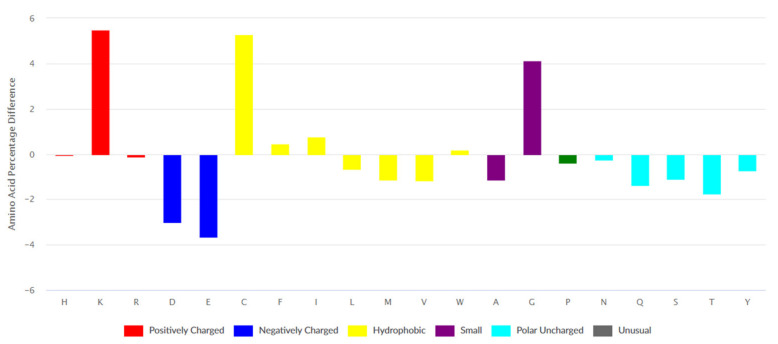
Amino acid composition of ribosomal peptides of DBAASP presenting as the difference from UniProt (https://dbaasp.org/statistics, accessed on 20 March 2021).

Data on 2568 ribosomal peptides was retrieved from the *DBAASP* database. The differences between amino acid compositions of AMPs’ ribosomal set from DBAASP and UniProt are presented in Figure 1. The peculiarity of the amino acid composition of AMPs is an abundance of bulky hydrophobic amino acids (Phe, Ile, and Trp) and also residues such as Cys, Gly, and Lys.

**Figure 2:**
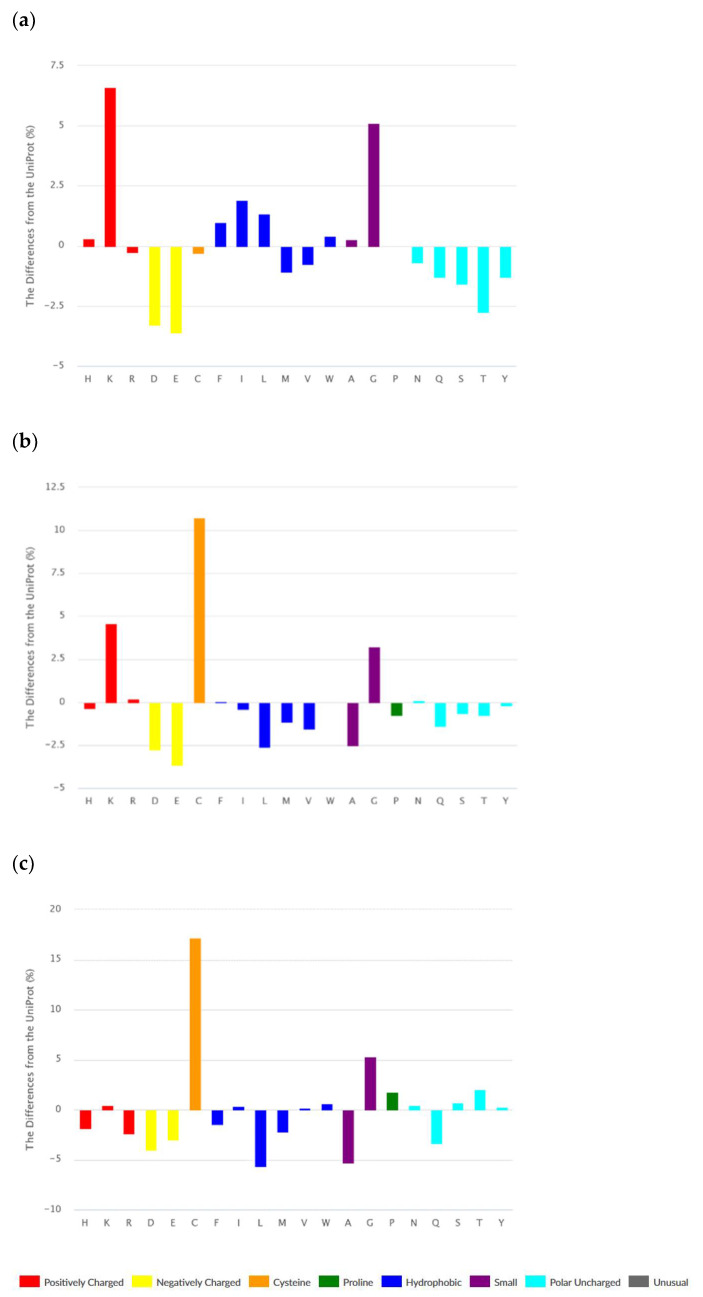
Amino acid composition of (**a**) linear; (**b**) disulfide-bonded, and (**c**) cyclic ribosomal peptides of DBAASP, presented as the difference from UniProt (https://dbaasp.org/statistics, accessed on 20 March 2021).

The set of ribosomal AMPs consist of linear peptides (1443), cyclic (N and C termini are covalently linked) peptides (123), and peptides containing intra-chain covalent bonds. The majority of peptides from the last class are disulfide-bonded peptides (1095). It is worth noting that many cyclic peptides contain disulfide bonds as well. According to Figure 2a–c, amino acid compositions of different classes of ribosomal peptides are distinguished from each other. At the same time, shared peculiarities are clearly seen between linear and disulfide-bonded peptides. The abundance of Lys and Gly, as well as the low level of acidic amino acids, can be seen as a common property.

It should be emphasized that the abundance of bulky hydrophobic amino acids (Phe, Ile, Leu, and Trp) and His is an intrinsic feature of linear AMPs only. We have to note that Phe, Trp, and His are aromatic at the same time. Cyclic and disulfide-bonded peptides are rich in Cys, Lys, and Gly. The hallmark of cyclic peptides is a high percentage of Pro, Ser, and Thr. The last fact can be explained by the requirement of many turns and bends to form the cyclic structure. Worth noting is that ribosomal cyclic peptides possess the least total positive charge among AMPs.

The results concerning the composition of two basic amino acids are very interesting. The portion of Lys in the sequences of ribosomal AMP is higher than it is at the average protein, while the portion of Arg is lower. AMP is mainly a cationic peptide and why their positive charge is mainly provided by Lys is a question to be answered. We will try to answer this question below when the differences in the mode of binding of the guanidinium group of Arg and the amino group of Lys with membrane are considered.

#### 3.1.2. Distribution along the Chain

**Figure 3:**
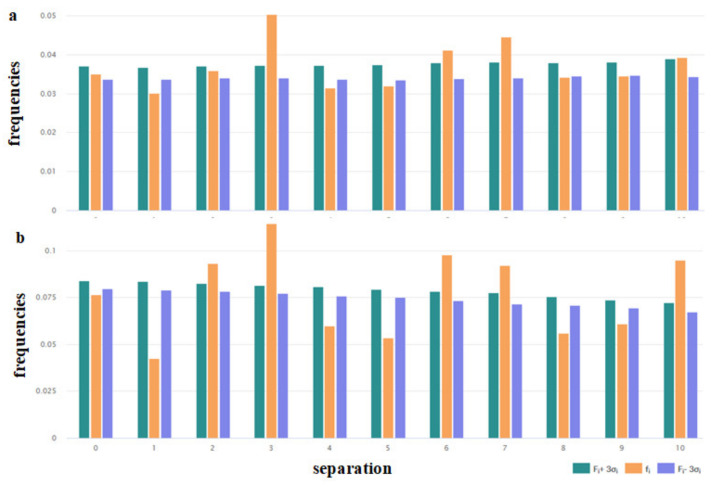
Frequencies of the appearance of pairs of (**a**) positively charged residues (R, K, H) and (**b**) hydrophobic residues (V, I, L, F, M) with the *i* residues (*i* = 0, 1, 2,…, 10) between them. *fi-* Observed frequencies; *Fi-* the average value of the frequencies assessed on the base of random sequences, and *σi* are their standard deviations. *Fi* frequencies are estimated for random sequences generated by shuffling of the sequences of the considered set of peptides. The assessments were performed using the tools of the page of “Statistics” (https://dbaasp.org/statistics, accessed on 20 March 2021) of the DBAASP [17].

Certain physicochemical features of peptides depend on the distribution of the hydrophobic, polar, aromatic, small, and other types of residues along the amino acid chain. To assess the peculiarities of the distribution of the key amino acids, the distribution of i-spaced amino acid pairs (DiSAAP) was estimated and compared with the same distributions in the randomly generated sequences. The distribution of DiSAAP for the basic (R, K, H) and hydrophobic (V, I, L, F, M) residues are presented in Figure 3a,b. Brown middle bars correspond to observed frequencies of DiSAAP *(fi)* in the set of ribosomal AMPs (derived from the *DBAASP* data [17]). The right, dark green and left, dark blue bars correspond to the values of *Fi + 3σ_i_* and *Fi − 3σ_i_* respectively, where *Fi* is the average value of the frequencies assessed on the basis of random sequences and *σ_i_* are their standard deviation (*i* = 0,1,2,…,10). Details of calculations of the distribution of i-spaced amino acid pairs are presented in the last paper on DBAASP [17]. Figure 3a,b show that the abundance of the pairs of basic amino acids with 3, 6, 7 residues between them (Figure 3a) and pairs of hydrophobic amino acids with 2, 3, 6, 7, 10 residues between them (Figure 3b) is not the result of random processes. Such distribution points towards the propensity of AMP to amphipathic alpha-helical conformation in the membrane environment.

**Figure 4:**
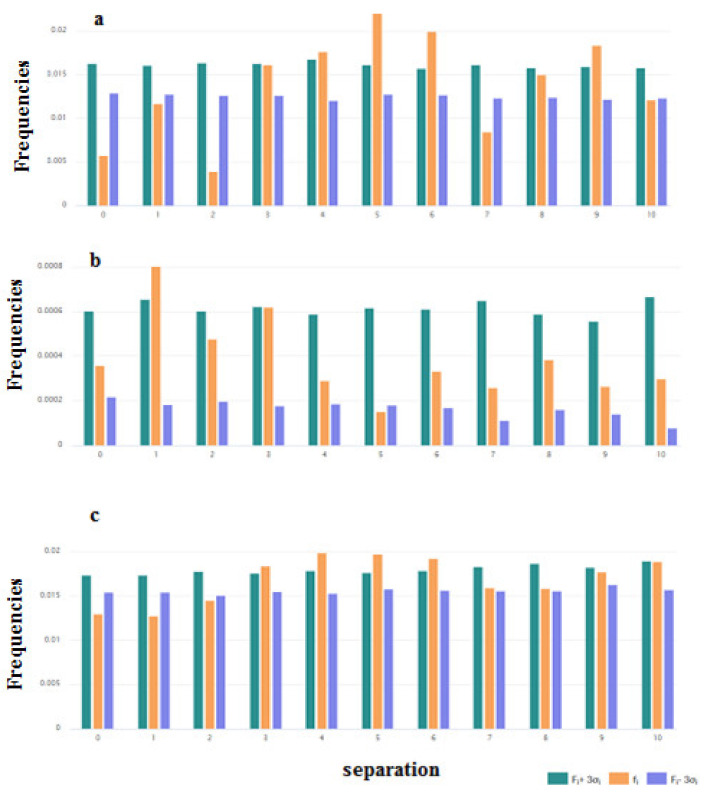
Frequencies of the appearance of pairs of amino acid with the *i* residues between them, for: (**a**) Cys (C) in the disulfide-bonded ribosomal AMPs set; (**b**) Trp (W) in the full set of ribosomal AMPs; (**c**) Gly (G) in the full set of ribosomal AMPs; The assessments have been performed using the tools of the page of “Statistics” (https://dbaasp.org/statistics, accessed on 20 March 2021) of the DBAASP [17].

It is interesting to consider the distribution of the pairs of key amino acids such as Cys, Trp, and Gly. These distributions are shown in Figure 4a–c. For the pairs of Cys, the distribution was built based on the data of disulfide-bonded AMPs (Figure 4a). From Figure 4a, it is clear that the results on the abundance of the pairs of Cys with the 4, 5, 6, and 9 residues between them are reliable. Such abundance can be connected with the fact that among disulfide-bonded AMPs more than 60% contain only one disulfide bond. The Cystines of such AMPs mainly form loops of about 6 to 9 amino acids long (including Cys), creating the hairpin or the structure of the shape of a “lasso” (disulfide ring is situated at one of the termini of the chain).

The preferred distance along the chain in the case of the pairs of Trp is one amino acid (Figure 4b). Aromatic Trp prefers to be in close proximity to each other in the sequences of ribosomal AMPs.

**Figure 5:**
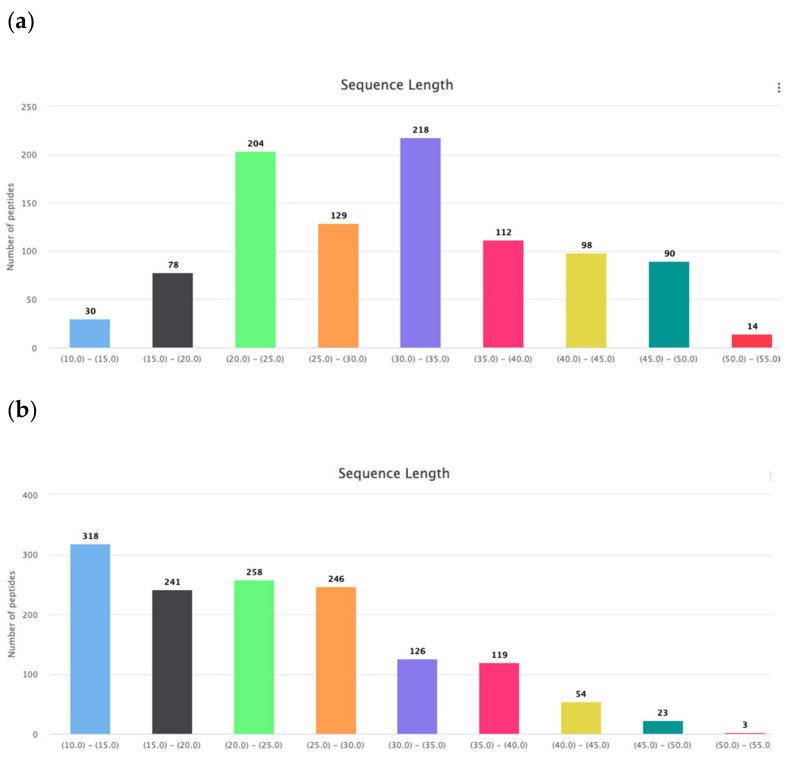
AMP’s lengths distributions for: (**a**) disulfide-bonded and (**b**) linear peptides (according to the data of *DBAASP* (https://dbaasp.org/statistics, accessed on 20 March 2021).

Gly is the amino acid with the most conformational freedom. Therefore their abundance in the AMPs can be linked with the necessity of conformational flexibility. It is considered that Gly, along with Pro, are responsible for the creation of turns and loops in the polypeptide chains to prepare conditions for the interactions between fragments of chain and stabilization of the tertiary structure. Therefore, it is easy to find a natural explanation for the abundance of Gly, Pro, Ser, and Thr in the cyclic AMPs, as was done above. Consequently, glycines facilitate the formation of a tertiary structure that can be stabilized by both non-valent interactions and intrachain covalent bonds (including disulfide bonds). Disulfide-bonded AMPs are longer than linear AMPs (Figure 5a,b) and their chains have to bend to form corresponding tertiary structure, and Gly, in this case, plays the appropriate role. The length of linear AMPs varies in the interval of 10–50 aa. The distribution of the AMP lengths points towards to the existence of two major groups of linear peptides: very short ones with the length 10–15 aa and short ones with length 17–30 aa. It has been shown that in the membrane-bound state majority of linear AMPs have the propensity to the alpha-helical conformation.

*Are the membrane-bound AMPs’ alpha-helices linear or curved?* This question is connected with the appearance of Gly and Pro in certain sites of the chain. It is considered that Gly, along with Pro, are responsible for the creation of kinks in the long alpha-helical fragments of both membrane and soluble proteins [19]. Comparing the portions of GLy + Pro residues in very short linear (length in the interval of 10–15 aa) ribosomal AMPs with the short (length in the interval of 17–30 aa) ones shows that percentage of GLy + Pro residues in the very short AMPs is lower (13%) then in short AMPs (17%).

**Figure 6:**
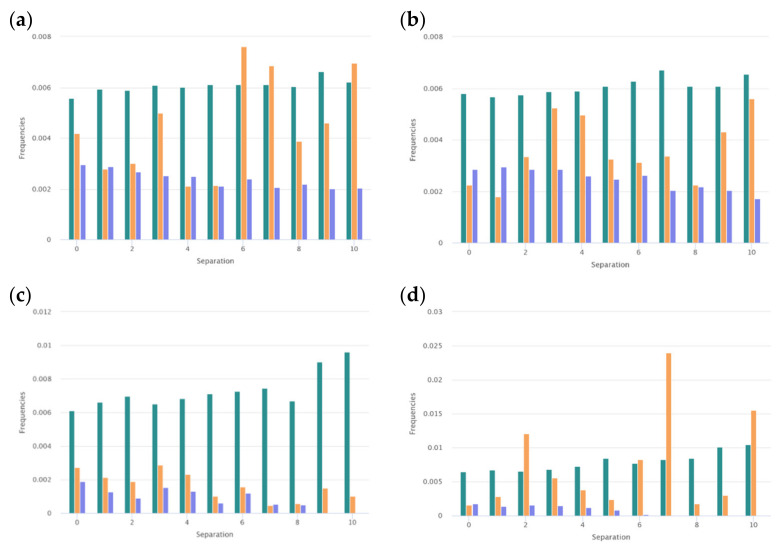
Frequencies of the appearance of pairs of amino acid with the *i* residues between them, for: (**a**) Gly-Pro in the set of short (17–30 aa) linear ribosomal AMPs; (**b**) Pro-Gly in the set of short (17–30 aa) linear ribosomal AMPs; (**c**) Gly-Pro in the set of very short (10–15 aa) linear ribosomal AMPs; (**d**) Pro-Gly in the set of very short (10–15 aa) linear ribosomal AMPs. The assessments have been performed using the tools of the page of “Statistics” (https://dbaasp.org/statistics, accessed on 20 March 2021) of the DBAASP [17].

It is interesting that the distributions of the pairs of Gly-Pro and Pro-Gly are also distinct for the very short and short AMPs. As shown in Figure 6, the abundance of the pairs of the Gly-Pro with the separation of 6, 7, and 10 amino acids points to the concentration of prolines in the direction of C -termini in short AMPs and so, on the convenience to create kinks. In contrast to this, the same distributions built for very short AMPs demonstrate that prolines are concentrated mainly at the N-termini to avoid the disruption of the helix.

At the same time, the distribution of pairs of Gly in the set of ribosomal peptides allows us to suppose that Gly’s function is to support the aggregates of AMPs. It is known that GxxxG motives in the alpha-helical fragments of the transmembrane proteins are responsible for the formation of alpha-helical associations in the membrane environment [20]. The results that indicate that the abundance of pairs of Gly with the distances of 3, 4, 5, and 6 residues along the chain are not random (Figure 4c) can be explained by demanding that the motives aggregate. However, the kinks can also be considered as supporting interactions between helices (resulting in the formation of helical aggregates).

To conclude, peculiarities of amino acid composition and their distribution along the chain allow the representation of AMPs as flexible peptides, not aggregative in the water environment, and having the potency to interact with the lipid bilayer (especially with microbial membrane) due to the abundance of the basic and aromatic residues. AMP sequences possess resources to adopt amphipathic alpha-helical conformation due to interaction with the membrane and resources to aggregate in the membrane environment.

## 4. Secondary Structure and Self-Aggregation

According to the 3D structure, AMPs are classified as alpha-helical, beta-structural, alpha +beta, unordered, etc. [3]. But this classification is conditional because the structure of many AMPs is determined by the environment and changes depending on the variation of environment and/or other factors (a local concentration of peptide, for instance). Moreover, AMP–membrane interactions are highly dynamic and biased towards changing and mutual adaptation of the peptide conformation to the particular membrane structure.

In membranes, the rules governing the formation of secondary structures and folds of the polypeptide chain are very different from the aqueous environment. In the membrane, hydrogen bonds become probably more important for driving secondary structure and forming aggregates in the membrane [21]. The hydrogen-bond effect helps to explain the easy formation of secondary structures in membranes. It is well known that each amino acid has its own propensity to a particular secondary structure. The propensity of individual amino acids to a particular secondary structure may be altered in response to the change in the environment from aqueous to the membrane.

In the aqueous environment, the bulky aromatic residues (Tyr, Phe, and Trp) and β-branched amino acids (Val, Ile) are favored to be found in β strands. Glycine is an intrinsically destabilizing residue in β sheets [22]. At the same time, a β-branched residue, such as Ile and Val, is described as α-helix destabilizing. Gly and Pro also destabilize the α-helices [22].

In the membrane environment, Ile and Val rank as the best “helix-promoters”, and it has been found that they are important for membrane protein assembly and folding [23]. Gly and Pro also display a considerable tendency to form α-helices in membrane environments [23]. The abundance of bulky amino acids Phe, Trp, Ile, and Leu in linear AMP tend to promote α-helix formation in the membrane [24] by interaction with aliphatic chains of the bilayer’s core, while at the more polar interface area, they can stabilize beta structural aggregates [25].

In the aqueous environment, bulky residues and positive charges can block alpha-helical self-aggregation. An abundance of positively charged Lys can also inhibit the formation of beta structure. Therefore the majority of linear cationic AMPs are disordered in the water environment and adopt regular secondary structure after interaction with membrane [24]. Here it has to be noted that AMPs rich in basic amino acids and/or prolines have a preference to ppII conformation [26]. Cyclic, as well as the majority of disulfide-bonded peptides, form a well-defined structure in solution. However, their structure in the membrane is membrane-dependent. For instance, PG-1 forms oligomeric transmembrane β-barrels in bacteriamimetic anionic lipid membranes, whereas in the cholesterol-rich membranes that mimic eukaryotic cells, the peptide forms β-sheet aggregates on the surface of the bilayer [27].

### 4.1. Secondary Structure

Amino acid composition allows making a supposition on secondary structure and propensity to aggregation of AMP. Secondary structure and propensity to aggregation along with the hydrophobicity and charge are essential determinants of antimicrobial potency. Peptide secondary structure and its propensity to aggregation depend on the environment. Consequently, AMPs, which are mainly linear, can adopt different conformations depending on the environment. The conformation of peptide GL13K is supposed to be disordered in water, α-helical in the zwitterionic lipid bilayer and beta-structural in an anionic lipid environment, especially when peptide to lipid ratio is high [25]. At high local concentration, the predisposition of peptides to self-aggregation into beta structure rises. Therefore, the GL13K−membrane interactions are governed by the equilibrium between the random coil, α-helical, and β-turn conformations. At high concentration, GL13K can initiate the β-sheet aggregates. Some other peptides (catestatin [28], cateslytin [29], fusion peptide of HIV [30]) similarly have exhibited varying equilibrium between α-helical and β-turn secondary structures depending on lipid composition.

Amyloid-forming peptides are the classic example of peptides that undergo a conformational transition from the native, mainly α-helical structure into an isoform with high beta-sheet content. They share key structural and functional features with AMPs [31]. Although some amyloid-forming peptides possess antimicrobial potency [32], they are well studied, mainly because they are considered to be immediate precursors for the formation of amyloid fibers and so are the most toxic components in many neurodegenerative diseases. To unravel the molecular interactions that occur during the transformation from alpha-helix to beta-sheet, the model peptide has been designed based on the well-studied α-helical coiled-coil folding motif [33]. Studies of the model peptide showed that the resulting secondary structure strongly depends on environmental parameters. It is worth noting that peculiarities of transition between secondary structures predetermine the velocity of formation and type of aggregates caused by self-assembling [25,33].

One more type of secondary structure that might be considered for AMPs is ppII. ppII conformation is convenient for peptides rich in basic amino acids and/or prolines. Buforine, a cell-penetrating peptide, due to propensity to the ppII conformation, finds its cytoplasmic target, DNA [34].

### 4.2. Self-Aggregation

Association of AMP with the lipid bilayer and creation of lipid–peptide complexes is a necessary step during the action on the microbial cell. Along with the ability to aggregate with lipids, a propensity to self-aggregation is crucial for the capability of AMP to affect microbial membrane [35].

Although not all AMPs can self-assemble, this property has been considered as an important property of peptides, because it can be influential for potency and mode of action. Peptides’ self-assembly is driven by electrostatic forces, hydrogen bonding, hydrophobic interactions, and π–π stacking interactions [36]. Consequently, a propensity to aggregation depends on environmental conditions.

Because AMP reaches target membranes through the aqueous phase, their properties in aqueous solutions are important for their effects on membranes. Rina Feder et al. tried to understand how AMP organization in an aqueous solution might affect the antimicrobial activity and concluded that potency correlated well with aggregation properties. They have shown that aggregation can have dramatic consequences on the antibacterial activity of the dermaseptin-derived peptides. Peptides that were more potent against bacteria were clearly less aggregated [37] in the aqueous environment. Furthermore, predictive models based on artificial neural networks indicate that peptide aggregation in solution indeed contributes to low antimicrobial activity [38]. Interestingly, the addition of cationic residues to peptides has been shown to inhibit aggregation in solution while improving antimicrobial potency at the same time [39].

Aggregation propensity is determined by sequence, and consequently, structural features of AMP. Linear AMP, because of its short length, non-high mean hydrophobicity, and relatively high net charge, exhibits disordered structure in aqueous solution and absence of aggregation (high net charge and the resulting electrostatic repulsion between peptides limits aggregation). Cycled main-chain or intra-chain covalent bonds represent a prerequisite for some structural stability of AMP in an aqueous solution and for the possibility of aggregation.

Self-assembling of linear peptides on the cell membrane can contribute to antimicrobial potency. It has been suggested that both in the case of pore formation or detergent-like mechanism, the process of the self-assembling of AMP takes place [16,40]. Recently, an attempt has been made to demonstrate a functional relationship between the AMP self-assembly and their antimicrobial activity [13]. As mentioned above, self-aggregation of cationic AMP is mainly governed by electrostatic interactions. Differences in the behavior of AMP depending on the environment (aqueous or membrane) are explained by the weakening of repulsion between positively charged groups in the membrane. An attempt [13] has been made to explore the behavior of designer peptides GL13K and their variants, including D-enantiomer at various pH of the solution, to model the process of repulsion weakening. The study of structural links between secondary structure, supramolecular self-assembly dynamics, and antimicrobial activity has been performed. It is not surprising that variation in pH and consequential evolution of secondary structures were related to a self-assembly process. It is interesting that the time of the initiation of self-assembly determines antimicrobial potency. For instance, two GL13K enantiomers formed analogous self-assembled structures, but D-GL13K initiated self-assembly faster and had notably higher antimicrobial potency than L-GL13K.

It is considered that the intrinsic antibacterial capabilities of peptide-based supramolecular assemblies have been largely overlooked and need more attention. The antibacterial activity of self-assembled diphenylalanine has been studied in order to gain insights into the significance of the interplay between self-assembly and antimicrobial activity [41]. It is worth noting that diphenylalanine is the central recognition module of the β-amyloid peptide. The study demonstrated that the interaction of diphenylalanine with bacteria causes damage to bacterial morphology, especially the membrane, and thereby inhibits bacterial growth. Non-assembled diphenylalanine samples, which consisted of the peptide at sub-critical concentrations, inhibited bacterial growth by 15–20% only. These results underline the significance of self-assembly for antimicrobial activity and allow us to conclude that the diphenylalanine motif can be used as a minimal self-assembling antimicrobial building block. Moreover, due to its low cost and high purity, it can serve as an important platform for the development of alternative antimicrobial agents and materials [41]. The hydrophobic and non-cationic nature of diphenylalanine makes it an attractive compound to combat resistance because, as it is well-known, several bacterial strains develop countermeasures mainly by modification of cell envelope, reducing the electrostatic attraction to the membrane.

It is worth noting that along with the hydrogen bonding, hydrophobic, and electrostatic Coulombic interactions, it has been suggested that self-assembly can be governed by aromatic π–π interactions as well (although the role of aromatic π–π interactions in peptide self-assembly is still the object of debate). Relying on the results of a study of model peptides Ac-(XKXK)(2)-NH(2), where X = Val, Ile, Phe, pentafluoro-Phe and cyclohexylalanine, it has been concluded that aromatic amino acids do not always more readily induce self-assembly relative to nonaromatic amino acids of similar hydrophobicity. At the same time, aromatic amino acids can create a unique morphology of fibrils [36].

Current knowledge on self-assembly mechanisms leads to the development of self-assembled antimicrobial nanomaterials and thus opens new avenues to tackle resistance problems [42]. AMPs consisting of charged, hydrophobic, and aromatic amino acids have a high potential to self-assemble and form fibrillar amyloid-like nanostructure or helical bundles in membrane environments; these structures allow them to exert their antimicrobial activity through an interaction with bacterial membranes [43,44]. The attention to the self-assembled nanostructures is explained by the appearance of the possibilities to solve several valuable problems such as increasing stability and half-life of peptides, rising and controlling the local concentration of the peptides enabling enhanced interactions [42].

Consequently, for some AMP, an interplay between self-assembly and secondary structure in the membrane environment is crucial for the action on the membrane and for the emergence of antimicrobial potency. At the same time, the modes of action of some other AMPs d not require self-aggregation and other properties predetermine their antimicrobial potency.

## 5. Frequently Occurred Amino Acids

Among AMPs the peptides rich of particular amino acids, such as Arg, Trp, Pro, Gly, Cys, and His occur. Modes of action of such peptides are determined by the physicochemical features of these residues. We will try to overview the knowledge on the behavior of such peptides in the membrane environment.

### 5.1. Basic Amino Acids and AMPs

It is known that AMP is a predominantly cationic peptide. But even among cationic AMPs, peptides with a high content of basic amino acids (with a percentage of basic amino acids 20–30% or more) appear. Such peptides are not self-aggregated in the polar (aqueous) environment, and it has been supposed that some of them (with a percentage of basic amino acids >50%) due to low amphipathicity cannot self-aggregated even in the membrane. Because of such extremes, it is reasonable to suppose for high cationic peptides (HCAMP) are peculiar and different from other AMPs’ mode of action.

#### 5.1.1. Lysine and Arginine in the AMPs

The mode of action of HCAMP is reasonable to consider in the light of data gained for cell-penetrating peptides (CPPs) because HCAMP and CPP shared physicochemical features and CPPs are membrane-active peptides also. The peptides: penetratin, Tat, Arg9, and R6/W3 are considered as CPPs and at the same time possess an antimicrobial potency [45]. Moreover, the energy-independent mechanism, named “direct translocation” has been fully characterized for the latter peptides. The Hallmark of CPPs is an abundance of basic (Arg and Lys) residues and/or Trp. Study of the internalization process of these peptides has shown that penetratin and R6/W3, which are amphipathic, preferentially interact with anionic regions of membrane, while non-amphipathic Arg9 and Tat interact only with anionic lipids. Interaction with anionic headgroups of lipids seem to be sufficient for Penetratin to trigger a helical conformation [46], which in water is unordered. However, for a higher content of anionic lipids, the α-helicity decreases in favor of β-sheet structures [47]. In contrast to penetratin, Tat and Arg9 remain unfolded even in the membrane environment. Therefore, it is supposed that “direct translocation” of R6/W3, and penetratin occurs when they are in a helical conformation, whereas Arg9 and Tat translocate as random coils. The studies of CPP translocation exhibit the crucial role of basic amino acids, especially Arg, for the last process. It has been shown, that translocation depends on the membrane potential also.

Although both Arg and Lys are basic amino acids with a high amphipathic index [48], they are differently interacting with cell membranes. The guanidinium group of Arg is a major cause of this distinction. It has been shown that the guanidinium group binds to the phosphate group of lipids more strongly than the amino group of Lys does [49]. Moreover, in the binding state, guanidinium group polarity is reduced and it possesses a capability to internalize membrane and to come into close contact with another guanidinium group to form a stacking contact [50,51]. These features of the guanidinium group provide the penetrating capability of Arg9 peptide, whereas Lys9 does not display a propensity to translocate across membranes [52]. Both Arg 9 and Lys 9 show anti-cooperative binding with membrane constituted of a mix of POPC and POPG lipids at low (up to 10%) POPG concentration, although Arg9′s anti-cooperativity is weaker. When the concentration of POPG rise up to 30%, Arg9 binding becomes non-cooperative, while Lys9′s binding remains anti-cooperative [49]. It has been shown that Arg9 can form stable complexes with an artificial membrane constituting zwitterionic lipid POPC, while Lys9 can not [49]. These peculiarities of side chains of Arg and Lys allow supposing that an abundance of Lys in ribosomal AMPs connected with the necessity to raise the selectivity of AMPs to the prokaryotic cell membranes. An abundance of Lys in ribosomal AMPs indicates a difference from them penetrating peptides’ modes of action for the majority of ribosomal AMPs, although Arg-rich peptides also appear among ribosomal AMP.

MD simulations of TAT peptides in the model membrane constituted of zwitterionic lipids showed that basic amino acids of cationic peptides try to move close to the phosphate group of lipids of both proximal and distal layers of the membrane [53]. At the high peptide to lipid ratio, attached to surface peptides produce a thinning of the membrane and so reduce the hydrophobic free-energy barrier to allow some side chains of basic amino acids to reach the phosphate group of the distal layer. The length of the lysine and arginine side chains facilitates to do this. As a charged group enter the hydrophobic lipid bilayer, water followed them. The water and phosphate penetration create transient pores and so allow some other peptides to diffuse across them [53]. It has to be noted that the Arg inserts into the membrane more easily than Lys [54]. As mentioned above, the special binding of the Arg to phosphate groups is explained by the specific structure of the guanidinium group.

Thus, electrostatic interaction can be considered as a driven force at CPP translocation across the membrane. Consequently, membrane potential has to affect the process of translocation. To explore the impact of membrane potential on CPP translocation, another MD simulation [55] has been performed for four types of peptides (WALP carrying zero, INLK carrying three, TAT carrying eight, and R9 carrying nine positive charges). Three lipid components, including DPPC, POPC, and cholesterol, were used to build a one-bilayer or two-bilayer model of the membrane. The local membrane potential has been produced by the ion concentration imbalance across the membrane or by external electric fields. Atomistic molecular dynamics simulations demonstrated that local membrane potential plays an essential role in translocating process. Due to the enhanced local membrane potential, CPPs readily enter cells through the opened membrane pores in a chain-like conformation. The penetration time was consistent with experimental data [55].

CPP is mainly cationic peptides, and we see that translocation across the hydrophobic part of the bilayer is not passive diffusion. CPP–membrane interactions cause some perturbations in the arrangements of lipids in the membrane. For the microbial membrane, where a portion of anionic lipids is relatively high and so attraction of peptides to the membrane is strengthened, more marked perturbations can be supposed. The prokaryotic membrane will promote an accomplishment of the high local concentration on their surface because it can provide the more sharp partition of peptides between the aqua and membrane. So, for HCAMP, the model that intends a perturbation of lipid bilayers with the creation of transient pores is even more acceptable in the case of the microbial membrane. Here, it is worth noting, that membrane potential, which is correlated with the translocation capability of HCAMP, is markedly higher in the case of the prokaryotic cell than eukaryotic.

Peptide to membrane binding can be the cause of structural changes of the membrane, and the carpet model is a widely used model describing the lipid packing defects induced by CPPs [45]; however, some other modifications induced by the CPPs are also considered. For instance, in the artificial membranes constituted of zwitterionic and anionic lipids (DMPC/DMPG) have shown that penetratin induces membrane invaginations resulting in the formation of tubular structures. Moreover, it has been demonstrated that membrane fluidity was crucial in the occurrence of membrane deformations after penetratin binding. In a membrane of fluid disordered phase (Ld), Penetratin is able to induce invaginations. At the same time, the peptide have an effect on a raft-like membrane [56]. So, exploration of the interaction of CPP with different membrane domains and revealing of the modifications of lipids’ organization are important for understanding the mechanisms of translocation. The influence of the CPP on the different microdomains of the plasma membranes with different phospholipid compositions has been investigated [57]. The results indicate that penetratin is able to induce rearrangements of membrane lipids that favor phase separation and membrane heterogeneity [58]. R6/W3 and Arg9 also have the capability to increase membrane fluidity that facilitates peptide translocation [58,59]. Recently new mechanism of transient pore formation has been presented for Arg9 [14]. The authors proposed that Arg9 translocate through the pores formed by means of membrane fusion.

#### 5.1.2. Histidines in the AMPs

Because the isolated histidine has a pK of approximately 6.5, it is largely unprotonated and uncharged at physiological pH while are protonated and cationic at acidic pH. So, histidine behaves as a basic amino acid at the acidic pH only. At the same time, histidine is classified as aromatic due to the presence of a sextet of p-electrons. Consequently, histidine can be involved in the different types of interactions: the coordinate interactions between histidine and metallic cations are the strongest, followed by the cation–π, hydrogen–π, and π–π stacking interactions [60]. When the histidine is in neutral form, the cation–π interactions are attractive; when it is protonated, the interactions become repulsive. The complicated nature of Histidine predetermines the pH-dependent mode of interaction of the His-rich peptides with the membranes [61]. At physiological pH, His -rich peptides behave differently from Arg- and Lys-rich peptides, while at the lower pH, they resemble Arg-rich peptides. For instance, histidine can form stable noncovalent complexes with acidic residues, including a phosphate group. These interactions have a similar chemical basis as in the case of Arg, although they are weaker [62]. Exploration of the empirical distribution of the protein-ligand cation–π interactions found in X-ray crystal structures shows that positively charged histidine residues are rarely involved in cation–π interactions, although the stacked π^+^–π interaction is estimated to be of similar magnitude to that of arginines [63]. By analogy to Arg, a protonated histidine has a propensity for forming like-charged contact pairs with another protonated histidine or with arginine [64].

A pK of the histidine side chain in the peptide depends on the local environment. It has been assessed that the pKs of histidine side chains of the SDS bound Hb-33–61peptide are of the order of 7.7 to 7.8 [65]. Hb-33–61 is a proteolytic product of the bovine hemoglobin alpha-chain and has potency against Gram-positive bacteria and fungi. It is supposed that small changes in the local pH may have large effects on the activity of histidine-containing AMP. For instance, the cationicity of the clavinines, other histidine-rich peptides produced by the solitary tunicate *Styela clava*, derives primarily from histidines, and they are active at pH 5.5 but relatively inactive at pH 7.4 [66]. Moronecidin, the 22 residue long peptide by analogy to the clavinine contains four histidines, but in addition, other basic residues also. The greater positive net charge of moronecidins accounts for the antimicrobial activity at neutral pH [67]. Consequently, the pH-responsive activity changes depend not only on the portion of histidines in the peptide but on the overall composition and position of the histidines in the chain. Histatin peptides belong to a family of salivary histidine-rich AMPs. Histatin-1 and histatin-3 are derived from the humans’ genes HTN1 and HTN3 [68]. Histatin-5 is a proteolitic fragment of the histatin-3. Despite the fact that histatins exhibit a high degree of sequence homology, histatins 1 and 3 ‘s activities are pH-dependent, while the activity of histatin 5 is pH-independent over the range of pHs 4 to 8. The differences in the behavior of histatins are explained by the acidic residues present in the carboxylterminal domains of histatins 1 and 3, which are absent in histatin 5 [69].

The mode of action of histatins is a subject of intense debate. It is supposed that all targets of histatins are intracellular. Translocation into cells may involve different uptake pathways (energy-dependent and energy-independent). There is a suggestion that once inside the fungal cells, histatin 5 affects mitochondrial functions [70]. As mentioned above, His-rich peptides bind copper (Cu) and other metal ions in vitro. Bis–His motifs are commonly found in biological systems that can form a coordination motif to create Cu(I)−bis-His complexes with O2 reactivity. This allows speculation that the Cu(I)−histatin complex could potentially mediate Cu-induced oxidative stress, which in turn may affect mitochondrial functions and could be the cause of the fungal killing. It is proved that Cu modulates histatins’ antifungal activity [71]. By analogy, the Zn^2+^ ions are modulated by the antimicrobial activity of clavanin A [72]. Zn^2+^-dependent antibacterial activities have been shown for other histidine–rich peptides [61], including histatin 5. It is worth noting that Zn^2+^ is considered to be functionally similar to the acidic pH, which imposes a positive charge on histidine-rich peptides [73]. It has been shown by confocal microscopy that Clavanin A-Zn2+ added to *Escherichia coli* translocate across the cell membrane to find a cytoplasmic target [72].

To their features, histidine is considered as a convenient residue to tune pH and cationic ion-sensing of the cell-penetrating sequences. The family of histidine-rich designer peptides, LAH4 is a well-studied example of such peptides. The development of LAH4 peptides was aimed at solving the transfection problem, but they possess antimicrobial activities also. Both processes, transfection, and antimicrobial actions require membrane-active sequences. For transfection, the peptide has to be involved in the trafficking to the endosome and when inside the cell, to provide an escape from the endosome. So the interaction of LAH4 peptides with the membrane was predominantly studied on the model vesicles mimicking plasmatic or endosomal membranes of the eukaryotic cells [74]. Both antimicrobial activities and endosomal membrane disruptive capabilities of LAH4 peptides are pH-dependent. Studies of action of LAH4 peptides on the vesicles mimicking the prokaryotic membrane, that is membrane constituted of anionic POPG lipids, showing that calcein release activity depends on pH and acyl chains’ saturation [75,76]. By adding additional residues being positively charged at the neutral pH, the HALO family of peptides active even at neutral pH has been created [77]. It should be noted that although the activity and pH selectivity of histidine-containing peptides are mainly determined by the number of histidines, peptides with the same number of histidine at different positions give neither the same activity nor the same pH sensitivity profiles [78]. Therefore, the pH-sensitivity of histidine-containing peptides can be tuned by manipulating histidine numbers and positions [79,80] to design agents that would function selectively in acidic compartments.

### 5.2. Aromatic Amino Acids and AMPs. Cation–π Interactions

It is known that aromatic systems interact strongly with cations. The side chains of Phe, Tyr, and Trp in the proteins are considered as a cation-binding site, so-called “hydrophobic anions” [81]. Because of the indole ring, tryptophane provides a much more intense region of negative electrostatic potential than does benzene or phenol, and so in proteins, it appears more intensively at the cation–π interaction sites. The complicated nature of Trp side chains predetermines its behavior in the membrane environment also. A membrane–Trp interaction is complex and governed by hydrophobic effect, dipolar, quadrupolar, H bonding, and cation–π interactions. The hydrophobic effect drives Trp out of the water, while complex electrostatic interactions push it to the headgroups of the lipids (to the hydrated interface of the membrane) [82]. The distribution of individual amino acids in known structures of helical membrane proteins has been studied, and propensities of their occurrence at different portions of the bilayer as a function of depth in the bilayer has been calculated [83]. The function that describes the behavior of Trp (a propensity to a particular membrane site) corresponds to Gaussian distribution with the global maximum at the distance of 11.9 Å from the middle plane of the membrane. Therefore, an estimation of how deeply a side chain of Trp prefers to penetrate into a membrane shows that the preferable depth of penetration corresponds to the interfacial region of the membrane. Sigmoidal curves describe the behavior of other bulky hydrophobic amino acids, such as Leu, Ile, and Val, showing that they penetrate into the membrane more deeply.

A high content (relative to average protein) of aromatic hydrophobic residues in the linear ribosomal AMPs allows suggesting an essential role in the functioning. Moreover, there is a group of AMP where Trp and Arg have a prevalence relative to other amino acids. The effort has been spent to understand the mode of behavior of Trp rich peptides in the membrane and to look for the causes of the differences from the peptides rich with other bulky hydrophobic amino acids. For instance, RW9 and RL9 peptides that have the same length and charge and similar hydrophobicity (according to the Eisenberg scale [84]) behave differently in the lipid bilayer [85]. RL9 is more deeply inserted into the membrane than RW9, though RW9 can translocate across the membrane in contrast to RL9.

AMPs predominantly are cationic peptides, and above we saw how Arg can drive the interaction of the peptide with the membrane. The abundance of Trp with its complicated nature put additional capabilities at the interaction of AMP with the membrane. Trp is considered hydrophobic due to its uncharged side chain, while it does not reside preferably in the hydrocarbon region of lipid bilayers [83]. Another important property of Trp is the extensive π–electron system of the aromatic indole sidechain that gives rise to a significant quadrupole moment [81]. Consequently, basic residues and Trp are capable of participating in cation–π interactions, thereby facilitating enhanced peptide–membrane interactions [86]). The cation–π interaction can take place in either a parallel (stacked) or a perpendicular orientation. In the stacked conformation, the Arg side chain is able to form the same amount of hydrogen bonds as when it is not involved in cation–π interactions [87]. This is in contrast to lysines, which cannot form hydrogen bonds while participated in cation–π interactions. The stacked arrangement between Arg and Trp residues is preferred in contrast to Lys and Trp. The cation–π interaction is possibly restraining the peptide structure in a suitable conformation to interact with the bacterial membrane. Arg and Trp-rich peptides can lead to structures that go far beyond regular α-helices and β-sheets. The Arg is effectively shielded from the highly hydrophobic nature of the bilayer by associating with a Trp residue when the peptides penetrate into the bilayer [81,86,88].

**Figure 7:**
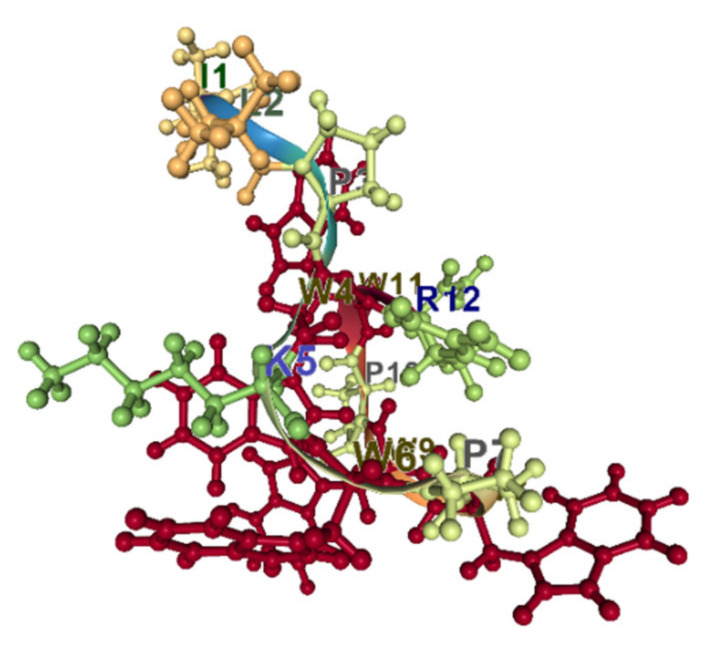
Representative structure constructed on the basis of MD trajectories simulated for the AMP Indolicidin (DBAASP [17] ID = 4807). Side chain heavy atoms are represented in ball-and-stick, and the backbone is represented in cartoon colored by residue index. Triptophane side chains are presented in red and basic amino acids side chains in green.

Indolicidin is a well-studied, short, 13 amino acid Trp-rich antimicrobial peptide. It belongs to the cathelicidin family of peptides. The proportion of Trp residues in indolicidin is the highest (about 40%). The peptide is unordered in water and adopts a wedge-type shape in the micelles [89]. Trp residues are segregated from positively charged ones and situated in a trough between positively charged regions (Figure 7). It has been shown that indolicidin can cross the membranes at concentrations above the MIC but below the minimal lytic concentration [90].

Antimicrobial peptides of the family of Lactoferricins that are known as Trp-rich peptides, show cell-permeable capabilities. Interacting with the membrane via electrostatic and/or hydrophobic interactions, they may form pores or inverted micelles to shuttle inside the cell [91]. Their mechanism to enter cells is similar to CPPs; once in the cell, they can interact with DNA or RNA, affecting their synthesis.

Puroindolines are small, cationic proteins and contain Trp-rich regions [92]. The mode of action of the 13-residue Trp–rich fragment of puroindoline, the puroA has been comprehensively studied by a variety of biophysical and biochemical methods [88]. In the bound to membrane state, all the positively charged residues are oriented close to the face of Trp indole rings. Due to the high content of Trp residues, puroA is located at the interfacial site of the membrane. The binding has an impact on the phase behavior of the vesicles. The amphipathic structure that appeared upon binding allows the peptide to insert more deeply into bacterial membranes and perturb the membrane bilayer structure [88]. The penetration of puroA into vesicles resembling bacterial membranes was more extensive than into vesicles mimicking the eukaryotic membrane.

### 5.3. Prolines and AMPs. ppII Conformation

Proline is α-helix breaking residue; helix kinks are a common feature of proteins and raise the conformational flexibility of the helical fragments [19]. It has been shown that proline residues in natural antimicrobial peptides define a hinged region that is crucial for antibacterial potency and selectivity [93,94].

When the portion of the Pro in the AMPs is very high, as in the Pro-rich antimicrobial peptides (PrAMPs), the mode of action does not involve the lysis of bacterial membranes but only penetration into susceptible cells, where PrAMPs act on intracellular targets [95]. PrAMPs are a group of cationic host defense peptides of vertebrates and invertebrates characterized by a high content of Pro-s, often associated with Arg-s in repeated motifs. PrAMPs show a similar mechanism and selectively kill bacteria, with low toxicity to animals.

Recently, a revised definition of the PrAMP as the class of AMP has been proposed [96]. According to the new definition PrAMP constitute peptides that satisfy the following set of criteria: have antibacterial activity; net charge >+1, proline contents >25%; key motif—PRP (indicative but not essential); have intracellular targets (for example, DnkA and/or 70S ribosome).

Drosocin, pyrrhocoricin, and apidaecin, representing the family of short (18–20 amino acids) Pro-rich antibacterial peptides, originally isolated from insects and act on a target bacterial protein chaperone DnaK [97]. The Pro-rich peptides have multiple functions and functional domains and perhaps carry separate modules for cell entry and bacterial killing. The Pro–Arg–Pro or similar motifs assist the entry into bacterial cells without any potential to destabilize the host cells, and therefore without toxicity to eukaryotes. The antibacterial activity of the native products is provided by the independently functioning active site, capable of binding to the bacterial DnaK and preventing chaperone-assisted protein folding [98]. Pro-rich cell penetration modules may be general for antibacterial peptides in nature. For instance, the cathelicidin hydrophobic tail sequences reveal strong similarities to C terminal tails of pyrrhocoricin, drosocin, or apidaecin [98].

The mode of translocation of PrAMP peptides is under debate. Proline-rich antimicrobial peptide Bac7(1-35) is a substrate of the SbmA, an inner membrane protein of Gram-negative bacteria. It is supposed that SbmA is involved in the internalization of Bac7(1-35) into the cell [99]. At the same time, although the monomer of the de novo designed PrAMP (Chex-Arg20) does not show membranolytic activity, dimeric, and tetrameric forms are membrane disruptive [100]. Consequently, it can be supposed that Chex-Arg20 possesses some penetrative membrane ability. The mode of translocation of PrAMP is determined by the details of amino acid sequence and conformational possibilities of particular peptides.

**Figure 8:**
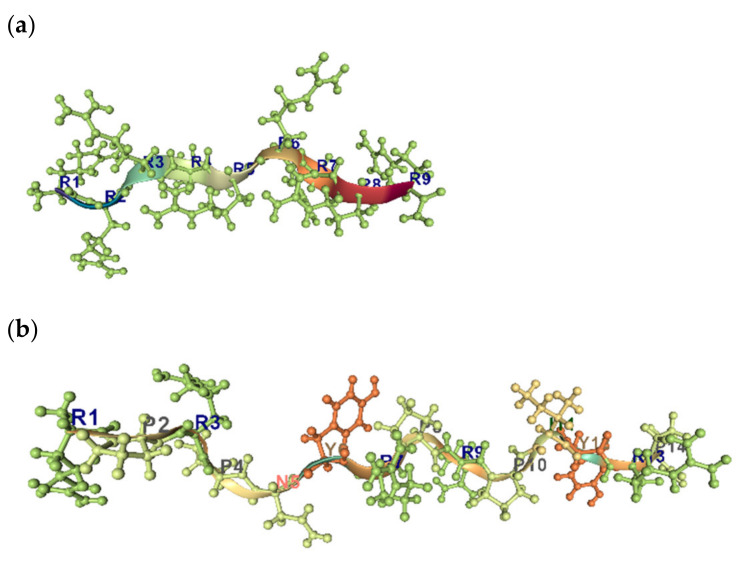
Representative structures constructed on the basis of MD trajectories simulated for the AMPs: (**a**) R9 (DBAASP [17] ID = 9015), and (**b**) Astacidin (DBAASP [17] ID = 2145). Side chain heavy atoms are represented in ball-and-stick, and the backbone is represented in cartoon color-coded by residue index.

Uversky et al. have shown that the combination of low mean hydrophobicity and relatively high net charge is an important prerequisite for the absence of stable structure in proteins under physiologic conditions, thus leading to “natively unfolded” proteins [101]. The view has been offered that unfolded peptides and proteins have a strong tendency to poly-proline II (PPII) conformation locally while conforming statistically to the overall dimensions of a statistical coil [26]. The PPII helix is often observed in the context of Pro-rich sequences, but sequences that are not enriched in Pro can adopt this structure also (Figure 8a,b). It has been show that Arg9 and TAT peptides, which are arginine-rich cell-penetrating peptides, adopt PPII type conformations in the membrane. By analogy, Pro- and Arg-rich peptides are also able to interact with biological membranes and form the transient pores, without lysis of cells [102].

It can be suggested that linear, cationic AMPs, which are disordered in aqueous solution and thought to be in a statistical coil state, may, in fact, be flickering in and out of a metastable PPII helical conformation. In the membrane environment, many of them form high ordered structures (alpha-helical, beta structured, or even aggregated) and so cause membrane disturbance and permeabilization. However, some of them, for example, Pro-rich peptides, do not behave as such, as they are not capable of forming high-order structures in the membrane and remain in ppII conformation in the latter environment. Proline induces the conformational flexibility of the polypeptide chain and so prevents a self-association of peptides. Flexibility is crucial for antibacterial potency and selectivity of many AMPs [94]. The proline residue in Buforin II, for instance, operates as a major translocation factor [103]. It should be noted that charged residues and Pro are the worst aggregators in both alpha and beta self-aggregation [104]. The pro-rich peptide is capable of aggregating only with conjugated to Gly form as it takes place in the case of collagens or other Pro/Gly rich polypeptides [105]. Collagen’s chains adopt conformation somewhat similar to ppII. Worth noting that ppII conformation and not *α*-helical is convenient to the aggregation with DNA [34].

### 5.4. Glycines and AMPs

Glycine is the amino acid with the highest conformational freedom. So their appearance in the sequence can be connected with the necessity to raise the flexibility of the peptide. Another hallmark of the Gly is a small side chain that facilitates the formation of the C^α^- H hydrogen bond. These two features promote the unexpected high content of Gly in the helical fragments of transmembrane proteins. Moreover, the majority of Gly from the transmembrane helix is situated in the hydrophobic area of the membrane and supports the stability of the complexes of the transmembrane helical fragments [106]. The support is expressed in the promotion of the inter-helical hydrogen bonds and/or by the creation of the kink in the helix. To create a pro-kink area, Gly frequently acts in a pair with Pro. Such a pair weakens the single helix stability, but at the same time, creates conditions for the inter-helical interactions and the formation of the stable helical complex [106]. Therefore, Gly plays an intriguing role in peptide/protein structure formed in the membrane environment where they can act as tightly packing amino acids with a flexible main chain. It means that Gly is the best “aggregator” residue.

In some AMPs, glycines are indeed considered as aggregators. Gly–xxx–Gly motives occur in several AMPs and they promote the self-aggregation or creation of the helical heterocomplexes. Studies of the amyloid-β peptide suggest that GxxxG glycine zipper motifs is responsible for dimerization and fibrillogenesis [107]. It is known that GxxxG motif mediates the helix packing. At the same time, this motif may also be critical for the formation and stability of the β-sheet structure. αβ peptide exists in a β-sheet or random coil configuration in the aqueous environment but converts to an α-helical structure upon membrane association [108]. The same transitions have been shown for other glycine zipper containing peptides, plasticins [109,110], and bombinins [111,112].

Plasticins and bombinins are members of Gly-rich peptides’ family. The family of Gly-rich antimicrobial peptides (GRAMP) unite the peptides widely distinct by sequence and functionality. Gly–Leu rich peptides, such as XT7 peptide from *Silurana tropicalis*, acanthoscurrins 1 and 2, leptoglycin, etc., are uncharged or weakly-charged linear peptides [113]. Armadillidins with the presence of a sixfold repeated motif GGGF(H/N)(R/S) are highly cationic linear AMPs [114]. While Gly–Cys rich peptides, such as ginsentides, are uncharged or weakly-charged but have a highly compact, pseudocyclic structure stabilized by disulfide bond network [115]. XT7 peptide, acanthoscurrins 1 and 2 and leptoglycin are active against Gram-negative bacteria and/or fungi [113]. Armadillidins have equivalent antimicrobial activity against Gram-positive bacteria, Gram-negative bacteria and filamentous fungi, but not against yeasts [114].

Consequently, it is difficult to suppose the common mode of action for GRAMPs. We can note is that flexibility is a requirement to make the linear peptide membrane active. Glycines mainly provide the flexibility of linear peptides. In other cases, glycines provide bends in the polypeptide chains to create conditions for the formation of the disulfide-bonds network to stabilize the certain structural scaffold and to form flexible loops with special motives.

### 5.5. Cysteines and AMPs: Intra-Chain Covalent Bonds

The majority of not-linear AMPs constitute peptides stabilized their structure by the disulfide-bond network. Many peptides share a structural scaffold but can have different functionality due to differences in amino acid sequences. The conserved scaffold of a tertiary structure at the sequence variations allows the molecules to show different surface distributions of polar and apolar residues and a variation in the cationicity. Often the AMPs are grouped according to a common scaffold and/or to a defined number of cysteines. For instance, peptides displaying a pattern of six-cysteines are united into one group and the peptides with the pattern of eight-cysteines into another. The ubiquitous class of AMPs named defensins can be presented as the three groups of peptides displaying three different patterns of six-, eight-, and ten- cysteines [116,117]. Correspondingly, scaffolds are stabilized by the network of three, four, and five disulfide bonds. Classical s AMPs are enriched in cysteine and glycine residues and stabilize the structural scaffold by the network of four disulfide bonds; however, there are other sub-families with 6 or 10 cysteines, also [118].

It is worth noting once more that the same scaffold does not mean the same functionality. For instance, both hevein-like peptides (8C-HLP) and ginsentides share a scaffold of eight cysteines that does not provide the same mode of activity of these peptides. 8C-HLPs have chitin-binding motives at inter-cysteine loop 3 and a conserved aromatic residue at loop 4, which are essential for chitin-binding and so, for antifungal activity [119]. Ginsentides fail in the ability to bind to chitin due to the absence of chitin-binding loops and so the spectrum of their targets is different from 8C-HLPs [115].

The structural scaffold of AMPs can be stabilized by other types of intrachain covalent bonds, such as, for instance, thioether bonds in lantibiotics. Amino acid lanthionine serves the same function as the disulfide bridges in defensins. For the lantibiotic nisin, the wedge model of action has been supposed [120] to be similar to the toroidal pore model. But later, it was shown that the nisins obviously use lipid II to bind specifically to the bacterial membrane, and the subsequent pore formation could proceed at much lower concentrations.

Oyster defensins have also been shown that they are specific inhibitors of a bacterial wall biosynthesis pathway rather than mere membrane-active agents; oyster defensin activity is a result of binding to lipid II [121]. So it can be supposed that a stabilized by intrachain-bond rings (loops) with the corresponding motives is a prerequisite for the binding to lipid II.

Two other bonds used for ring formation in AMPs are lactam and lactone bonds. A lactone ring is generated by cyclization of the C-terminal carboxylic acid with the side chain of serine or threonine, while cyclization between the C-terminal carboxylic acid or acidic side chains and the side chain of lysine or ornithine forms a lactam ring structure—AMPs with such intrachain bonds mainly synthesized by bacterial or fungal sources. Most antimicrobial cyclic peptides affect the integrity of the cell envelope [122].

It is clear that not linear peptides are more structured than linear ones due to stabilizing their tertiary scaffold by the intrachain-bonds network. Their flexibility is mainly linked with loops. So if loops possess corresponding motifs, the interactions of AMPs with each other or with other molecules (saccharides, proteins, lipids, etc.) can be more specific. Consequently, we can say, that a mode of action of not linear AMPs mainly determined by the organization of the surfaces of the conservative scaffolds, for instance, by the peculiarities of amino acid sequences of the loops.

## 6. Modes of AMP Interaction with Biological Membrane

In the coarse-grained approximation, the interaction of AMP with membrane can be concluded by two kinds of results: permeabilization of the membrane and/or penetration through it to reach an intracellular target without membrane disruption [2].

### 6.1. Permeabilization

The studies of the permeabilization of the membrane aimed at understanding the modes of action of AMP have taken place over several decades and have relied on theoretical and experimental approaches. MD simulations are mainly used as the theoretical approach [123,124,125]. The experiments were mainly performed on artificial membrane structures, such as large unilamellar vesicles (LUV) or giant unilamellar vesicles (GUV), using different biophysical methods, which mainly looked at the leaking capabilities of vesicles [126,127,128,129]. During the early stages of studies, it has been suggested that most AMPs cause membrane permeabilization through one of three possible routes: carpet, barrel-stave pore, or toroidal pore [130,131]. A detailed survey of these mechanisms has been presented in several reviews [63,132,133]. Despite a huge amount of work, a consensus in understanding of AMP action is still lacking. The formation of membrane-spanning pores is experimentally proved only for Alamethicin [134,135] and lytic toxins [136]. These classical pore-forming peptides might be the exceptions. 

Consequently, the question remains open: *What are the mechanisms behind all-or-none and graded leakage?* [137]. In many cases, leakage can be explained by transient-pores formation [125]. Peptide-caused perturbations in the lipid arrangement leading to phase transitions and the appearance of defects in the membrane have been considered as the reason for these temporal not-structured pores. For instance, for magainin 2, a mechanism of action has been suggested that implies high tension at the external monolayer of the membrane, caused by increasing its area by means of insertion of amphipathic peptides into it. An impact of such tension on the internal monolayer can become the reason for the rapture of the monolayer and can lead to events forming pores stochastically. The radius of pores depends on magainin 2 concentration [129]. The experimental investigation of the mechanism of the release of the contents of phospholipid vesicles induced by another AMP, cecropin A, allowed to propose the model of pore-formation, an alternative to the conventional one [127]. This model does not require oligomerization of peptides and suggests a disorganized structure of the pores, which are not neatly lined by peptides. Even in the porous state of the vesicles, the fraction of peptides are retained on the membrane surface. Therefore, according to the authors of [127], cecropin A may cause membrane thinning and positive curvature strain, opening up pores that allow a complete release of contents. An unstable porous state of the vesicles is relaxed by lipid transport through the transient pores.

Consequently, there are views that other mechanisms that do not involve the perforation of the membrane may exist. Results obtained from differential scanning calorimetry, nuclear magnetic resonance, and freeze-fracture microscopy studies [29,138] show that clustering of the cationic antimicrobial peptides with certain anionic lipids can be the cause of the membrane crowding. These and other works have suggested that phase separation and/or domain formation may be an alternative mechanism of action for certain antimicrobial peptides [11]. Atom force microscopy study of the small cationic AMP revealed that they induce the formation of cardiolipin-rich domains with a concomitant reduction in the ordering of the lipid acyl tails [126,128]. This remodeling effect results in structural instabilities in the model membranes, suggesting phase separation as an alternative mechanism of antimicrobial peptide action. So there is increasing evidence that the efficacy of some cationic antimicrobial agents is determined by their effects on membrane domains [139,140]. That is, AMPs target specific membrane components and can induce specific restructuring of the membrane [141]. The specific restructuring of the membrane can be the cause of the delocalization of peripheral membrane proteins impacting energy metabolism and/or cell-wall biosynthesis [142]. Here, it could be noted that restructuring of the membrane is not only the basis of the new mechanisms that do not involve perforation, but it also is a main theme in the conventional (old) models of AMP activity [143]. It can be assumed that preferential clustering of the cationic peptide with certain anionic lipids also provides an environment necessary to achieve the conventional modes of membrane permeabilization [130].

To uncover pore-formation mechanisms at an atomic level, the MD simulations of the systems “peptide + bilipid” have been performed. The 21-residue AMP, isolated from the skin of the green-eyed tree frog, allowed, in the bilayer of phosphatidylcholine lipids, to develop a pore-forming mechanism distinct from some proposed models where AMP insertion was proposed via large surface aggregates [123]. Maculatin-induced membrane leakage has been visualized experimentally [144]. MD simulations show that maculatin, initially resides in the state parallel to the surface and individual peptides subsequently adopt transmembrane (TM) orientation. An energetic barrier for maculatin TM-insertion is overcome by cooperative actions, involving two peptides in a head-to-tail arrangement in combination with a water defect. At equilibrium, peptides are continually changing between marginally stable TM oligomeric assemblies and surface-bound states on both interfaces [125]. The authors concluded that: pores form by consecutive addition of individual helices to a transmembrane helix or helix bundle; Maculatin forms an ensemble of structurally diverse temporarily functional low-oligomeric pores; these pores continuously form and dissociate in the membrane.

The modern knowledge of the mechanisms of membrane permeabilization induced by AMP lead to the question: are there any common mechanisms at the interaction of the AMP with the membrane, followed leakage? or are there many different mechanisms depending on physicochemical features of AMP, composition of membranes and their state? The result of the interaction of AMP with the membrane in the majority of cases is leakage, which takes place either through stable pores or through unstable, unstructured, transient pores. The formation of stable pores has been proven experimentally for several AMPs only. Leakage induced by the majority of AMP is explained by transient defects (pores) in the membrane. We would like to note that in the current models, the transient pore formation is the cooperative process and often is accompanied by translocation of the part of peptides either to the internal monolayer of the membrane or into the cytoplasm [41]. Translocation into cytoplasm allows AMP to get intracellular, negatively charged targets (such as DNA, RNA, proteins, and mitochondrial membrane in the case of fungi and cancer cells, etc.)

### 6.2. Translocation

For many AMPs, pore formation capabilities depend on their concentration. For instance, PGLa at high concentration can only form pores, while at low concentration, it adopts a well-defined surface-bound S-state [145]. Other examples are CPPs, which at high enough concentration perturb membranes and make them permeabilized [9], and cecropin A, which at low concentration may reach cytoplasmic targets before the membrane permeabilization [146]. Moreover, for highly charged AMPs, the formation of the classical pore may not have to be considered at all to explain peptide translocation and membrane permeabilization. Peptide-lined pores are not needed at all to explain how a highly charged peptide can translocate across a lipid bilayer. Instead, simple cooperative effects involving 2–3 peptides can be used to explain translocation [124]. Indeed, it has been shown that some CPPs with large fractions of cationic residues are able to silently translocate membranes without causing too much leakage [14,147,148].

Therefore it is reasonable to suppose that processes of translocation and permeabilization of the membrane by peptides share some common events, and both processes are characterized by cooperativity of peptides’ actions on the membrane. Recently, it has been shown that some AMPs possess both capabilities: they can translocate through the membrane and simultaneously form transient pores. As mentioned above, for some of these peptides the result of interaction with membrane depends on the concentration of peptides [9,145,146]. But in the case of Pur A, both results, translocation and permeabilization, take place at the same peptide concentrations. It has been shown that peptide PuroA simultaneously passes through and creates pores in the membrane, although these two events are shifted in time [15]. All of these means that part of peptides pass through the plasma membrane before the pores are formed. The results of several separate events (particular interactions) have to be determined by the relations between the kinetics of the events and the time necessary to gather enough actors for cooperative action. In the case of PuroA, results are determined by the kinetics of the passing through the membrane and by the kinetics of the creation of transient aggregates for the pore-formation.

Recently, a new mechanism of penetration, different from those previously proposed, that is from directly passing through a lipid membrane, has been presented for Arg rich peptide [14]. Experiments on eukaryotic cells and membrane vesicles mimicking eukaryotic membranes have allowed concluding that passive entry of Arg-rich CPPs takes place via branching and layering of membranes, followed by fusion.

Consequently, passive cell penetration mechanisms, especially for Arg-rich peptides, have not been unraveled yet and future studies are required to understand the molecular details of the CPP-membrane interactions.

### 6.3. Cooperation among AMPs: Synergism

Mode of action of particular AMP entails sequential interactions of the peptide with the lipid bilayer and with other peptides. The results of these interactions might be aggregation of the peptide with others or just the creation of conditions for others to perform their mission easily [53]. For example, according to the mechanism of action thought to be relevant for maculatin, binding and insertion of a particular peptide into the membrane lead to lowering of the energy barrier of TM-insertion for other peptides [125]. In this context, it is clear that the mode and results of the interaction are peptide concentration-dependent [9,145]. Consequently, in many models of AMP action, the interactions of the peptides with lipids and with each other provide conditions that are crucial for getting the final goal. Moreover, nature has provided a defense system for the organisms by various AMPs, which act according to their own modes of action and have certain potency, but at the same time can interact with each other and are strengthening the total potency [149]. The latter fact presents an additional argument to the statement that the ways to oppose the development of resistance are well-optimized by the defense system based on the AMPs [4].

The strategy of fighting against the development of resistance used by nature becomes attractive to combat the resistance of microbes against previously active antibiotics. Current strategies to overcome the problem of resistance to conventional antibiotics do not only use certain AMPs as a killer of multi-drug resistant bacteria, but they also use them together with conventional antibiotics. The supposition about the success of combination therapy is supported by the facts that combination of drugs potentially eliminate resistant strains, delay the evolution of drug resistance, reduce the dosage of individual drugs, and hence, diminish side effects [150,151,152]. Recent studies report that success depends on the results of the combination, which can be synergistic or antagonistic [153,154]. Synergistic drug pairs can efficiently kill bacteria but intensify the selection of resistance, while antagonistic drug pairs showed the reverse trends. Therefore, knowledge of the results of the interaction of combined drugs is required.

Studies of the synergy of AMPs and conventional antibiotics to reveal more effective combinations have been intensively expanding [155,156,157,158,159]. The results of the investigations have been widely surveyed [160]. Here we will overview the knowledge concerning synergy between AMPs only, and will present conjectures concerning the mechanisms of the synergistic actions.

An exploration of the pharmacodynamics of six different AMPs (cecropin A, LL 19–27, melittin, pexiganan, indolicidin, and apidaecin) by testing their individual and combined effects in vitro, allowed the conclusion that the synergy is a common phenomenon in AMP interactions [161]. It is worth noting that three-AMP combinations are even more synergistic than two-AMP combinations [161]. Moreover, for *Xenopus laevis* [162], *Tenebrio molitor* [163] and bumble bee *Bombus terrestris* [149], it has been shown that producing AMP cocktails is an efficient way to combat bacterial invasion.

The phenomenon of synergy was explored in AMPs of the Temporin family from the skin of *Rana temporaria*. Temporins A and B are active against Gram-positive bacteria but not against Gram-negative ones, while Temporin C is active against both groups of bacteria. It has been supposed that resistance of Gram-negative strains against Temporins A and B is linked with the capability of peptides to oligomerize in the presence of the outer membrane’s LPS [164]. Oligomerization hinders moving towards the inner membrane. It is interesting that Temporin C can prevent oligomerization of Temporins A and B promoted by the outer membrane. Therefore, when Temporins A and B were mixed with temporin C, a marked synergism was observed [164].

PGLa and magainin 2 (MAG2) are the most well-studied AMPs from frog skin that show synergistic antimicrobial activity. The molecular mechanism of synergy between the last peptides was studied on the atomistic level with the help of MD simulations in [165], as well as experimentally by different biophysical and biochemical methods [166,167,168,169].

In the early work of Matsuzaki and coworkers, they supposed that the synergistic action of magainin 2 and PGLa was a consequence of the formation of the heterodimeric complex [166]. The complex was characterized by fast formation and moderate stability. It has been shown that the complex was not formed in the aqueous phase. Each peptide separately binds to the membrane and the complex is formed in the bilayer. Formation of the complex promotes the shift of the partitioning equilibrium of each component toward enhanced binding. Therefore, it has been supposed that the synergism is partially connected with the increased binding and formation of the heteromolecular complex [166]. In another work [168], it has been suggested that the orientation of AMP relative to a lipid bilayer in the bound state predetermines the mode of action. Two states of AMP are considered: surface (“S-state”) and transmembrane (inserted “I-state”). For the given membrane with particular curvature, the peptide state is concentration-dependent [168]. According to the heterodimer hypothesis mentioned above, it was proposed that PGLa and MAG2 formed a complex, where PGLa adopts transmembrane orientation, while MAG2 stays on the bilayer surface and stabilizes water-filled pore by interaction with PGLa [167].

To look for the amino acids necessary for synergy and to reveal details of molecular interactions, the mutating variants of PGLa and MAG2 were explored [169]. Zerweck and coworkers used three different experimental approaches to explore the impact of amino acids at a certain position of the sequences on the different appearances of synergy. Checker-board assays, allowing assessment of the level of activity of the combined action of mutated variants of the PGLa and MAG2. With the help of the solid-state 15N-NMR study, the orientation of peptides relative to the lipid bilayer surface at the combined action was assessed. Leakage experiments gave assessments of the sizes and stabilities of the defects in the lipid bilayer created by PGLa and MAG2 in cooperation. This multi-approach study allowed to reveal the GxxxG motif in the PGLa as necessary for synergy. The motif is located between the polar and hydrophobic faces of the helical wheel. It is interesting that GxxxG motifs are important for molecular contacts, but not just the motif, the exact position of the motifs in the sequence is crucial as well. In conclusion, the authors developed a molecular model of the functionally active PGLa-MAG2 complex, where Gly-Gly contact allows PGLa to form the membrane-inserted antiparallel dimer [169]. The role of MAG2 is to promote the membrane-inserted state of PGLa and to stabilize the tetrameric heteropeptide complex. According to the model, the PGLa monomer is in contact with one MAG2 molecule at its C-terminus. Electrostatic interactions between anionic groups in MAG2 and cationic residues in PGLa are the basis of the latter contact. Here, it is worth noting is that the study of the synergistic effects for other peptides derived by mutation allowed the conclusion that although electrostatic interactions enhance synergy, they are not necessary for the synergistic effect [169].

Along with the biophysical methods, molecular modeling and computer simulations are widely used to understand the behavior of the AMP in the membrane environment [165,170,171,172]. Many efforts to describe the atomic level interactions taking place in the system composed of peptides and lipids have been made. It is worth noting that the limited computational resources do not allow to perform a comprehensive description of complex systems composed of many molecules of lipids and peptides. Nevertheless, all-atom molecular dynamics simulations give valuable information about the details of the interactions of molecules. Recently, the results of 5–9 μs all-atom molecular dynamics simulations of MAG2 and PGLa in DMPC or 3:1 DMPC/DMPG membranes have been reported [165]. The work has been aimed at investigating pore formation and stabilization processes. Because of the limitations in computational resources and unknown time scale for pore formation, the simulations started from tetrameric helical bundles inserted in a transmembrane orientation. The rationale was that these starting conditions correspond to the local free energy minima of the pore-forming systems. Due to the fact that transmembrane state as starting for MAG2 is not acceptable [167] and it has been shown that the starting arrangement has an impact on the final structure of the pore (complex) [165], we have to emphasize that the above simulations are questionable in the case of MAG2 homotetramer or heterotetramer of MAG2 -PGLa. Here it is worth noting that because of computational limitations, peptide to lipid molar ratio (P/L) in this simulation [165] was equal or less than 1:30. It is known that at such high peptide concentration, PGLa has the propensity to adopt transmembrane, I –orientation [167]. Consequently, starting the simulation from inserted-into-membrane tetrameric helical bundles can be rational in the case of PGLa. However, the same is not correct for MAG2 up to P/L 1:10 [167]. Apparently, this is the reason why PGLa tetrameric assembly is stable nearly for the full MD trajectory and involves four monomers in tilted T-states. At the same time, the simulation on the Mag2 tetramer shows that two of the monomers adopt S state orientations on opposite leaflets during the first microsecond [165]. Nonetheless, MD simulation allows imagining the mechanism of synergy between MAG2 and PGLa. For instance, interactions between residues S8 and E19 in MAG2 and K12 and K19 in PGLa, make the antiparallel heterodimer more stable than the homodimers [165]. MD simulation at the atomistic level shows that E19 of MAG2 are indeed essential for maintaining the synergistic effect.

The synergistic effect of MAG2 on the insertion and aggregation of PGLa has been assessed in coarse-grained molecular dynamics simulations [170]. In these MD experiments, the lipid bilayer contained dilauroylglycerophosphocholine (DLPC) lipids and the peptide/lipid molar ratio equaled about 0.023. The helical structure of peptides was fixed and not changed. At the initial stage of the simulation, peptides were placed above the equilibrated bilayer surface with different heterodimeric orientations (parallel or antiparallel). Simulations showed that the synergistic effect from MAG2 promotes tilting, insertion, and aggregation of PGLa. It has been indicated that PGLa aggregates only in the presence of MAG2, and not in the system without MAG2 [170]. The authors supposed that MAG2 form parallel heterodimers with PGLa and induce the aggregation of heterodimers in the membrane. MAG2 tends to interact with the bilayer surface, while PGLa is tilted and inserted into the hydrophobic region of the bilayer to lead to pore formation [170].

Consequently, it can be concluded that when magainin 2/PGLa mixtures are studied in fully saturated lipid bilayers, magainin remains oriented along the membrane surface, whereas PGLa adopts transmembrane alignments. At the same time, it should be noted that biological membranes mainly consist of unsaturated lipids. It has been shown that in membranes that carry unsaturations, both peptides are aligned along the bilayer surface [173]. Consequently, a mechanism for synergistic antibacterial activities should necessarily consider the last fact.

## 7. Concluding Remarks on the Grouping of AMP

Many attempts have been made to design compounds against microbes relying on the features of AMP, which are responsible for the mode of action [174,175]. Due to difficulties with determining features responsible for the particular mode of action, as well as the absence of quantitative description of these mechanisms, designing has been mainly performed experimentally and has relied on the known active peptide sequences and on reasonable substitutions of the amino acids at the certain positions of the parent peptide [176,177,178]. In these cases, design has been coupled with structure-activity relationship studies. Another approach that has been used is an in silico study, relying on machine learning on the sets of data on experimentally explored peptides [179,180,181,182,183]. The aim is to uncover the features of peptides that are most informative in distinguishing one type of peptides from another and based on which an efficient predictive model can be established. Current predictive tools mainly offer a binary classification of peptides (into antimicrobial and not antimicrobial) [181,182,183,184]. For the target-oriented design, finer classification of peptides is required—one that would take into consideration the capability of an intra-AMP grouping of peptides, for instance, according to results of interactions of AMP with envelope. The latter interactions can lead to different results. The results of actions that always start by binding of AMP with the outer layer of the envelope may be the following: (a) inhibition of the vital pathways in the outer layer of envelopes (for instance, inhibition of the transferring of the cargo for peptidoglycan synthesis by Lipid II [185]; (b) permeabilization of the plasma membrane (for instance by disruption of the membrane through pore-formation or carpet-formation [130,131]); (c) perturbation of plasma membrane structure without unrecoverable changes (for instance, perturbation of the raft proteins and corresponding biological pathways through interaction with lipid microdomains [142]); (d) translocation through the plasma membrane (without unrecoverable changes) and inhibition of metabolic pathways in the cytoplasm [103,186]. It is worth noting that the intra-AMP grouping according to the results of interaction with envelope is conditional. There are the peptides whose interaction with the envelope gives several different results simultaneously. For instance, it has been shown that peptide *PuroA* simultaneously passes through the envelope and creates pores in the membrane [15].

There have been many different efforts to perform the intra-AMP grouping to raise the efficiency of the design. The intra-AMP grouping has been tried based on the data on sequences, structures, target organisms, source organisms, etc. The design relied on many different encodings of sequence or structure. Taking into consideration the high variability of AMPs in the particular organism and often their multifunctionality, the target-organism-based [187] and source-organism-based [188] groupings could not be effective for the de novo designing.

Predictions based exclusively on sequences as strings of letters and relying on the different machine-learning and the so-called linguistic approaches are widely used for de novo design [183,189,190]. Sequence-based binary classification models are detail-ignoring approaches, however, because they aim to uncover common Grammar in the set of AMPs which unites groups of peptides with different modes of action and therefore with different Grammars. Moreover, approaches based only on sequence are anyway accompanied by loss of information, valuable for the accurate description of the interactions with the membrane. If the entire 20-letter alphabet is used, the data on the similarity of amino acids (between Lys and Arg, for instance) is lost. In this case, when similar amino acids are grouped and a simplified (short) alphabet is used, the loss of information (for instance, on differences in physicochemical features of Lys and Arg) takes place as well.

The grouping of AMPs according to 3D structure distinguishes alpha-helical, beta-structural, alpha/beta, unstructured peptides, etc. The majority of AMPs are linear and can adopt drastically different conformations depending on the environment. For instance, the beta-amyloid peptide is disordered in the water environment, creates alpha-helical oligomers in the membrane at low local concentration, but at high local concentration, peptides are self-assembled into beta-structural complex [191]. Another example is cationic plasticine, which has shown multiple conformational transitions, including destabilized helix states, beta-structures, and disordered states [192]. Although tertiary structures of intra-chain-bonded peptides have a more certain topology, it has been shown that a similar tertiary scaffold does not mean similar functionality [115]. Consequently, the structural classification of AMPs is conditional and ambiguous.

**Figure 9:**
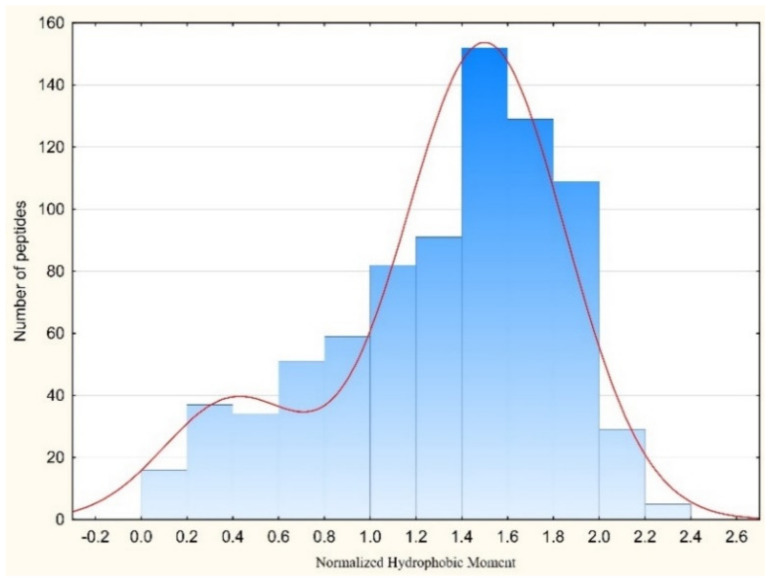
Distribution of the normalized hydrophobic moment (µ) of short (8–23 aa), linear, ribosomal AMPs using the tools of the page of “Statistics” (https://dbaasp.org/statistics, accessed on 20 March 2021) of the DBAASP [17]. The fitting has done by the sum of two Gaussian curve (red).

**Figure 10:**
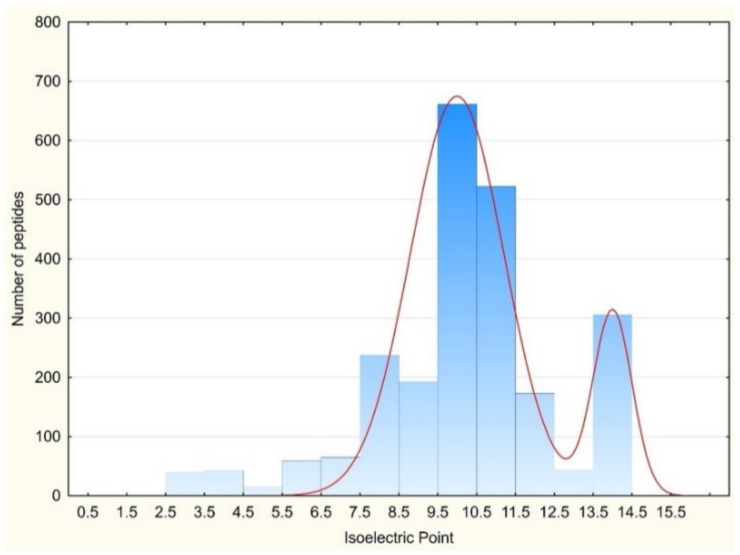
Distribution of the Isoelectric Point of ribosomal AMPs using the tools of the page of “Statistics” (https://dbaasp.org/statistics, accessed on 20 March 2021) of the DBAASP [17]. The fitting has done by the sum of two Gaussian curve (red).

In any case, the mode of action of AMP and the results of this action are determined by the physicochemical properties (PCP) of peptide and the composition of the cell envelope. Interactions between AMP and envelope can be described by simple physical forces, although these interactions may lead to a wide spectrum of outcomes. Current data allow to unambiguously distinguish AMPs according to their physicochemical features. There are peptides with a high value of total positive charge and peptides with zero or even negative charges; peptides with high hydrophobic moment and peptides which are not amphipathic (Figure 9); peptides with the isoelectric point around the value of 14 and peptides with a lower isoelectric point (Figure 10). Therefore, it is reasonable to think about the grouping of AMPs according to physicochemical features and to assign a particular mode of action for each group of peptides [180]. A clusterization of peptides according to physicochemical features has recently been performed [193,194]. Results of clusterization in these works allow to conclude that AMPs can be grouped according to physicochemical features, that is, the physicochemical space of AMP is discontinuous. At the same time, due to the small size of the available sets of AMPs, the results can not be considered as fully reliable. Therefore, the question *is the physicochemical space of AMPs discrete?* is still open. To answer this question, more data on peptides that are active against a particular strain is required.

Therefore, when aiming to develop a predictive model with high performance, it is reasonable to encode peptides based on their physicochemical properties (PCP) that are responsible for the interaction with cell envelope, and to try to perform classification using these properties as features. At the same time, during classification, we have to remember that particular peptide with certain physicochemical properties (PCP) can interact differently with different envelopes. Here the question arises: *what is an optimal set of physicochemical descriptors that should be used?* The authors of [174,180] have used many different descriptors to classify AMP such as charge, hydrophobicity, hydrophobic moment, a propensity to disordered structure, etc. We have to emphasize that proper attention has not been paid to very important physical interactions, such as the cation–π interactions. A comprehensive understanding of the role of the latter interaction in the functioning of AMP is crucial, for instance, to answer the following question: *what is a cause of necessity of abundance of aromatic residues (including Trp) in the AMPs? and why does nature preferably use Lys and not Arg when designing AMPs, given that it has been shown that Arg can enhance either the membrane permeability or translocating capability of AMP* [195].? As mentioned above, Lys and Arg interact differently with cell membranes and aromatic residues. Guanidinium group of Arg is the major cause of this distinction. Special binding of Arg to phosphate groups of lipids or rings of aromatic residues is explained by the specific structure of the guanidinium group [49]. Because Arg is considered as an important residue to rise activity, there is further motivation to increase Arg content in the de novo designed AMPs [195]. Due to the last tendency, a portion of Arg in synthetic peptides is remarkably higher than in ribosomal peptides (see “statistics” page of DBAASP https://dbaasp.org/statistics, accessed on 20 March 2021).

In this work, we have supposed that the abundance of Lys in ribosomal AMPs is connected with the necessity to raise the selectivity of AMPs to the prokaryotic cell membrane. But this is only a conjecture, and future studies of the role of Arg and Lys in the interactions with lipid phosphate or headgroups and aromatic residues of amino acids are necessary to understand the modes of action more comprehensively and to develop efficient models of in silico design of AMP. To our best knowledge, descriptors/features that reflect the peculiarity of Arg and Lys in light of cation–π interactions have not been used in previous AMP classification models. Consequently, it is fair to say that the optimal classifier has not yet been developed, and the process of looking for the optimal set of descriptors to classify AMPs should be continued.
